# Towards the Recognition of the Emotions of People with Visual Disabilities through Brain–Computer Interfaces

**DOI:** 10.3390/s19112620

**Published:** 2019-06-09

**Authors:** Jesús Leonardo López-Hernández, Israel González-Carrasco, José Luis López-Cuadrado, Belén Ruiz-Mezcua

**Affiliations:** Computer Science Department, Universidad Carlos III de Madrid, Av. Universidad 30, 28911 Leganés, Madrid, Spain; 100381018@alumnos.uc3m.es (J.L.L.-H.); jllopez@inf.uc3m.es (J.L.L.-C.); bruiz@inf.uc3m.es (B.R.-M.)

**Keywords:** affective computing, brain–computer interfaces, signal processing, visual disability

## Abstract

A brain–computer interface is an alternative for communication between people and computers, through the acquisition and analysis of brain signals. Research related to this field has focused on serving people with different types of motor, visual or auditory disabilities. On the other hand, affective computing studies and extracts information about the emotional state of a person in certain situations, an important aspect for the interaction between people and the computer. In particular, this manuscript considers people with visual disabilities and their need for personalized systems that prioritize their disability and the degree that affects them. In this article, a review of the state of the techniques is presented, where the importance of the study of the emotions of people with visual disabilities, and the possibility of representing those emotions through a brain–computer interface and affective computing, are discussed. Finally, the authors propose a framework to study and evaluate the possibility of representing and interpreting the emotions of people with visual disabilities for improving their experience with the use of technology and their integration into today’s society.

## 1. Introduction

In essence, a brain–computer interface (BCI) is a system that aims to read the activity of the human brain, thought to be the most complex biological system in the world [1]. Through a BCI an individual with a disability can have effective control over devices and computers, speech synthesizers, assistance applications and neuronal prostheses [2]. Currently, there are different research studies that focus on brain signals as a central point for assisting people with disabilities, considering it viable to analyze brain signals to convert these signals into instructions that are executed by external devices or to interpret people’s emotions [3]. Assistive technologies for people with disabilities are of great interest, and in fact, there are numerous studies aimed at improving their quality of life. However, it is still difficult to access assistive technologies, because usually they are only focused on one type of disability [4,5]. Most BCI research presents mental tasks and paradigms related to visual stimulation which then later analyze brain signals [6]. 

This work evaluates different research of the last decade, from 2009 to 2018, based on the application of the BCI for people with disabilities. The main search included BCI for people with visual or motor disabilities and the detection of affective states or emotions in people with visual disabilities. The research identifies an area that can still be explored, which is discussed throughout this manuscript.

For the realization of this work, the authors considered the recommendations and guidelines of Brereton et al. described in [7] and by Kitchenham et al. proposed in [8] for a systematic review of the literature. 

**Figure 1:**
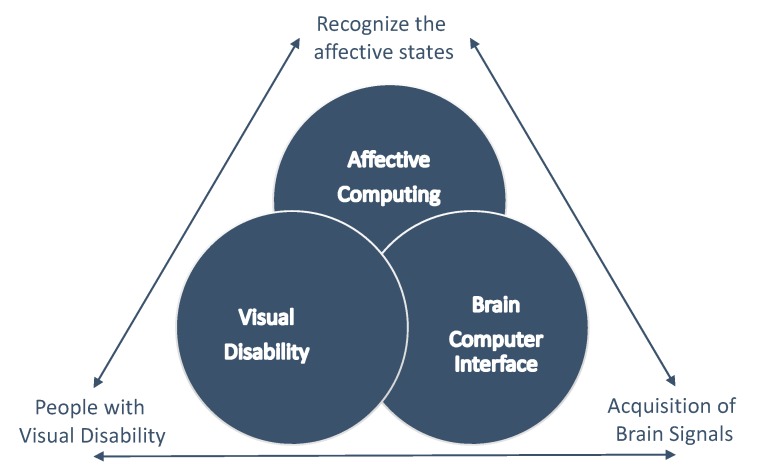
Topics evaluated in this work.

In Figure 1, different areas of interest for this research are presented: BCI, affective computing (AC) and visually disability. As shown in Figure 1, all areas together are relevant for the acquisition of brain signals through a BCI, to recognize the affective states of people with a visual disability using AC.

The following questions are a fundamental part of this research:

1. Why is it important to study the emotions of a person with a visual disability?
2. Can artificial intelligence through affective computing obtain information of interest to represent the emotions of a person with a visual disability?

The answer to these questions is exposed in the discussion section.

The most common way to identify an emotion is through facial expressions and speech, although these expressions are not commonly available in all situations. In some cases bio-signals are required to examine the emotional state [9]. Accordingly, the analysis of the emotions of a person with a visual disability through a BCI and current technological tools could lead to improvement in their quality of life and integration into society.

In [10], a pilot system for communication between the brain and computer was proposed, based on evoked potentials (EP), which served as the basis for the BCI. In recent years, the study of BCI has grown exponentially [11], where the main objective was to provide a channel of communication between an individual and a computer through the analysis of brain signals.

Current data show that the efforts made have been developed with the implementation of BCI systems, seeking to improve people’s quality of life. In [2,12], they classify BCI into seven groups, according to the neural mechanisms and recording technology used. The continuous advancement of technology and its inclusion in people’s lives is resulting in improvements in accessing technologies and also new forms of communication between people and things [13]. 

The goal of this work was defined as follows: firstly, the evaluation of BCI systems and its impact on the lives of people with a disability, and secondly the integration of BCI and AC in the detection of emotions to people with visual disabilities. The results obtained from the analysis are detailed in the discussion section.

The text is organized as follows: Section 2 shows a review of the technologies for the implementation of BCI systems and those related to AC and visual disabilities. Section 3 presents an analysis of related research with this work. Section 4 compares the different studies presented in Section 3, and in the Section 5, possible challenges in the research of systems that integrate BCI and AC for people with visual disabilities are presented. Finally, in Section 6, conclusions and future work are presented.

## 2. Perspective

In this section a general description of brain–computer interfaces, affective computing and visual disability is provided.

### 2.1. Brain–Computer Interface

A BCI involves the work of the brain and a device that is shared to enable a communication channel between the brain and an object that is controlled externally, as described by Prasat et al. [14]. Their study describes the implementation of the classification of the movement of the left hand and the right, through a BCI. 

Lebedev et al. in [15] proposed a two-fold classification of BCI: invasive and non-invasive. The first types are implanted at the level of the brain (intracranially) and their goal is to obtain signals of the highest quality. At the same time, the non-invasive ones that are placed on the scalp are based on electroencephalogram (EEG) recordings of the surface of the head. 

In [16], Minguillon et al. determined that EEG recordings are generally contaminated with noise generated during the acquisition of the signals, which can be caused by endogenous reasons (physiological sources such as eye, muscle and/or cardiac activity) or exogenous ones (non-physiological sources, such as impedance mismatch, coupling of power lines, etc.). 

A method for the extraction of characteristics without noise is proposed in [17]. The results obtained by Jafarifarmand et al. demonstrate the effectiveness in the extraction of the desired EEG characteristics. 

In [18], Arvaneh et al. proposed an algorithm for EEG channel selection. The proposed algorithm is formulated as an optimization problem to select the least number of channels within a constraint of classification accuracy. As such, the proposed approach can be customized to yield the best classification accuracy by removing the noisy and irrelevant channels.

In [19], Kübler et al. exemplified the objective of a BCI, providing people a tool to interact with a computer without the need for any muscular participation.

The evoked potential stimuli are related to the electrophysiological measurements of the processes that have to do with certain cognitive functions of the brain (attention given when performing an activity) [20]. 

The evoked potentials (EP) are identified by fluctuations of the electrical potentials of the brain in a cognitive process caused either by the occurrence of an event or the presentation of a stimulus [21]. With reference to the polarity, the components of the EP can be of two types: positive P, positive polarity and negative N, negative polarity. The P300 is a type of positive EP, which appears at 300 ms after the presentation of a stimulus or event, and has proven to be one of the main approaches of the BCI to provide an effective communication channel [22]. 

The P300 evoked potential occurs after the start of the stimulus, which can be physical, visual or auditory. EEG and EP techniques have been used to evaluate brain activity (brain functions) and sensory function. However, EP related to events have not been used regularly [23].

In [24], electrodes that do not require gel or even a direct coupling of the scalp have been considered for practices of the BCI. This study compares wet electrodes with dry and non-contacting electrodes within a BCI paradigm of visual evoked potential. They present the development of a new capacitive electrode, without contact, that uses a custom integrated high impedance analog front-end. The contactless electrode data, which work on the upper part of the hair, show 100% accuracy compared to wet electrodes.

### 2.2. Affective Computing

Part of human interaction involves expressing emotions, which can be through speech or facial expressions [25]. AC is considered a discipline of artificial intelligence which seeks to develop computational methods oriented to recognizing human emotions, in addition to generating artificial emotions using computers. Emotions are a psychophysical response to an external stimulus. People express their emotions based on communication with other people [26]. In an attempt to capture the emotions of a person through a computer and the need to improve the interaction between people and the computer. Picard [27] established the main concepts of affective computing and its relationship with people with disabilities.

### 2.3. Visual Disability

A visual disability is a condition that directly affects the perception of images, whether partially or totally. Vison is a global sense that allows us to identify objects at a distance and at the same time. A visual disability is related to visual acuity and visual field. The term visual disability is used when there is a significant decrease in visual acuity even with the use of glasses, or a significant decrease in the visual field. People with some degree of visual disability must make a greater effort to interact with the world around them and to thereby achieve social inclusion [28]. 

## 3. Related Work

This section considers different research related with the implementation of BCI for people with disabilities. The study performed divides the research into those focused on people with visual or motor disabilities and those that integrate BCI and AC for detection of emotions for people with visual disabilities. Although not all of these technologies were identified together in a single investigation, they were considered separately as part of this review, because they include criteria related to the main search—BCI and disability or BCI and affective computing.

### 3.1. BCI for People with a Visual Disability

In an effort to make a BCI usable for people with a visual disability, in [19] the authors included a BCI that was adapted to auditory stimulation. The proposal consisted of presenting letters of the alphabet in a 5 × 6 matrix. The individual must first choose the row and then the column. The results showed that the subjects obtained a performance above the probability level. The considerations of the experiment indicate that the accuracy of the spelling was significantly lower compared to a BCI system of visual stimulation.

A non-invasive BCI based on EEG for people with a visual disability proposes the conversion of captured moving images through a camera and converting them into visual signals for the optic nerve as a concept of a dnon-invasive artificial vision system [29].

The BCI based on visual mobility has been shown to be highly effective and is widely used. However, for patients who have vision problems or lose control of their eye movements, the possibility of interacting with a BCI based on vision is limited. 

Guo et al. investigated a brain computer auditory interface using the mental response [30]. The research proposed the use of auditory stimuli that allows a person to mentally select a target between a random sequence of spoken digits. The reported results indicated an average accuracy of 85% with five trials.

In [31], Hinterberger et al. proposed a BCI called “Thought Translation Device”, which operates with the voluntary response to auditory stimuli (auditory instructions) and feedback. One of their cited objectives was to provide a new tool to people with visual disabilities. For the experiment, three groups of people were trained to be stimulated in visual, auditory and visual/auditory combined. The results showed an average of 67% correct responses in the visual condition, 59% in the auditory condition and 57% in the combined condition. Although the results indicated that the visual stimulation was slightly higher, the research assumed that a BCI with auditory stimulation could be used for communication between the brain and a person with a visual disability.

An exploratory study of the viability of an auditory BCI is presented in [32] by Nijboer et al. Sixteen healthy volunteers participated in the training that consisted of thirty tests, lasting from two to three minutes. In those experiments, the increase or decrease of sensorimotor rhythms was achieved. Half of the participants were stimulated with visual stimuli and the other half with auditory stimuli in order to evaluate the evoked potentials of the affective state and the motivation that were considered in each session. The results showed that, although the performance of the visually stimulated participants was greater than the auditory stimuli, with enough training time an auditory BCI could be as efficient as a visual BCI. In addition, the viability of an auditory BCI has been investigated in a few studies using different EEG input signals, for example P300 evoked potentials [32]. The evaluations contemplated for this point were aimed at helping people with a visual disability.

Klobassa et al. in [33] indicated that people with severe disabilities or visual limitations require auditory BCI. This research studied whether six environmental sounds were useful to operate a P300 speller. The results of the analysis showed that the participants of the experiment achieved a precision score between 50% and 75%.

### 3.2. BCI for People with Disabilities

The implementation of BCI systems to analyze P300 visual evoked potentials in people with motor disabilities (progressive muscular problems that produce physical disability) are still being studied. 

In [22], the authors worked with a group of people and their possible interaction with a BCI based on P300. The results indicated specific technical data on the EEG channels and the frequencies for obtaining and analyzing the P300.

The case study implemented in [34], showed that a person with a severe motor disability was able to use a non-invasive BCI system for communicating messages with his family. This system is based on visual evoked potentials, taking as reference the P300, for the evaluation of a spelling module. The results indicate that the use of an BCI system can result in benefits for people with severe motor disabilities.

A research study revealed the effectiveness of a BCI based on P300 for a group of people (eight individuals with severe motor disability problems and eight healthy individuals without disabilities). BCI was operated successfully by both groups of individuals and the results indicated a non-significant difference in terms of the operation of the BCI [35].

In [36], a portable non-invasive BCI was presented to move a mobile robot in a home environment and operate a virtual keyboard. The results showed two participants successfully handling a robot between several rooms, while other participants managed to write messages with a virtual keyboard. They also observed that one of the volunteers was a person with physical disabilities who suffered from spinal muscular atrophy (severe motor disability).

An experiment involving a BCI based on EEG and for supporting people with disabilities is described in [37]. The BCI implements the concept of EP through P300 waves and N2PC (Evoked Potential with a negative deviation in the waveform that occurs approximately 200 ms after the stimulus is presented). The authors developed three applications: the first was an internet browser, the second was an application that controls a robotic arm and the third, was an application that allows people with severe disabilities to use basic commands related to emotions and their needs.

Motivated by the specific problems experienced by people who are paralyzed (severe motor disability), in [38], Hill et al. described a BCI that stimulates auditory in a group of people. The results indicated that the users modulated the brain signals in a single trial, which allowed the conscious direction of the attention with enough assertiveness to be useful in a BCI system.

The development and testing of a BCI based on the study of an EEG that was intended for use by completely paralyzed people was reported in [39]. The participants were stimulated in an auditory way. The group consisted of 13 individuals, of which the results showed a score between 76% and 96% for the task of choosing left and right. Hill et al. considered auditory EP to be a competent technique for the development of communication systems in people with disabilities.

Suwa et al. in [40] presented a new paradigm of BCI that uses the P300 and P100 responses, which occur in the frontal lobe and the temporal lobe, respectively; they used these responses stimulated by an audio in a single task. The main advantage of a designed paradigm is to get two different types of responses in a single EEG test task.

To improve the performance of the BCI, Yin et al. in [41] proposed a bimodal BCI approach that simultaneously uses auditory and tactile stimuli. The proposed BCI was an independent vision system because visual interaction of the user was not required.

An invasive BCI was developed for the neurological control of a high-performance prosthesis. Exposed by Collinger et al. in [42] the authors implanted two 96-channel intracortical microelectrodes in the motor cortex of a 52-year-old with tetraplegia. The participant was able to move the prosthetic limb freely in the three-dimensional workspace.

Moving a BCI from the laboratory to real-life applications still presents challenges. The objective of [43] was to integrate a mobile and wireless EEG and a signal processing platform based on a cellular phone in a portable and wireless BCI. The results of this study showed that the performance of the proposed cell phone-based platform was comparable, in terms of the rate of information transfer, with other BCI systems.

### 3.3. BCI for Detection of Emotions

A system of music generation according to the state of affectivity of a person was presented by Daly et al. in [44]. This proposal contemplated a BCI for acquiring an EEG for visualization and analysis of brain signals, a module for detecting the affective state of a person and a set of rules that allowed the system to generate music. Together with the BCI that detects the emotions of a person, in [45] the authors developed a system for generating music that served as a support for musical stimulation with short pieces. 

In [46], the evaluation of emotions was presented using electroencephalogram (EEG) signals. The linear classifiers were used to classify discrete emotions (happiness, surprise, fear, disgust and a neutral state). Audiovisual stimulation was used to evoke the emotions. The evaluated results represented a possibility to determine the emotional changes of the human mind through EEG signals.

Miranda et al. [47] presented a new type of BCI: the brain–computer musical interface (BCMI). The study mentions three principal problems: extracting information from significant control of signals emanating directly from the brain, designing generative musical techniques that respond to information and training subjects to use the system. A BCMI test system was implemented that used electroencephalogram information to generate music online. Likewise, it was mentioned that other research based on a better understanding of brain activity associated with music cognition and the development of new tools and techniques for implementing generative music systems controlled by the brain, point to a bright future for the development of BCMI.

In [9] Hamdi et al. implemented a BCI system and a sensor that measures heart rate, to identify the six basic emotions proposed by Ekman (anger, disgust, fear, joy, sadness and surprise). The results revealed that it was possible to identify the emotional state of the person.

Khosrowabadi et al. in [48] presented a system for the detection of emotions based on EEG. This system uses a self-organized map for the detection of the limits of emotions. The characteristics of the EEG signals are classified considering the emotional responses of the subjects, using the SAM (self-assessment maniki) study and their scores. The audiovisual stimuli that were used reflected the results of the proposed method in improving the accuracy to 84.5%.

An affective BCI was described in [49], based on an exploratory study for the modality of an affective response. The case of 24 subjects and their neurophysiological responses during visual, auditory and audiovisual stimulation were analyzed. The results showed that during visual stimulation the alpha parietal power signals decrease, while they increase during auditory stimulation. 

In [50], they described the recognition of emotions through an EEG as a field of computation with problems related to the induction of emotions, the extraction of characteristics and their classification. In addition, they present a characteristic extraction technique with a concept called the mirror neuron system that was adapted for the induction of emotions through the process of imitation.

In order to find the relationships between EEG signals and human emotions, in [51] Nie et al. studied brain signals, which were used to classify two types of positive and negative emotions. Results with an average test accuracy of 87.53% were obtained.

In [52], they mentioned the emotional recognition of objects as one of the research topics for continued work. They also observed that recognition and classification of musical emotions is still difficult. They used and EEG by means of a non-invasive BCI, to analyze brain signals, and finally proposed a personalized model, based on evidence for the recognition of the emotion of music.

Studies on the relationship between emotions and musical stimuli that use an EEG are increasing. Byun et al. investigated the characteristics for the EEG pattern classifiers, related to musical stimuli [53]. Feature extraction methods were applied with a database for the analysis of emotions. For future work the authors mentioned classifying the emotional state according to the music listened to.

In [54,55] Liu and Sourina used electroencephalograms to make more intuitive interfaces. Their research included the development of different affective applications, emotional games and emotional avatars. The authors implemented an algorithm of recognition of emotions in real-time. The results indicated that the algorithm was able to recognize eight emotions with good precision. 

In a study by Tseng et al. [56], a brain computer interface-based smart multimedia controller was proposed to select music in different situations according to the user’s physiological state. 

The study conducted by Xu et al. in [56] analyzed whether the performance of an auditory BCI can be further improved by increasing the mental efforts associated with the execution of the attention-related task. 

Koelstra et al. presented a database for the analysis of emotions using psychological signals with a set of data for the analysis of the affective states of a human [57]. The classification was performed for the scales of arousal, valence and liking using features extracted from the EEG and other modalities. The results were shown to be significantly better than random classification.

The main research performed in this work includes different studies specifically related to BCI for the detection of emotions in people with visual disabilities. Although not all the found technologies were integrated in a single research, they were considered as part of this review. To the best of our knowledge, the results do not show evidence of the integration of BCI and AC for detection of emotions in people with visual disabilities. For this reason, development of new research which integrates these topics of interest is proposed in this area of opportunity. 

## 4. Results

In this section a comparative analysis of the reviewed works is presented. It is observed that there is a trend towards the creation of BCI systems based on EEG, as a support technology for people with disabilities. In addition, it is possible to visualize the combination between BCI and AC systems, where the results of this analysis indicate that this combination is possible. The authors also analyze general purpose studies, that is, BCI for experimental research and its behavior with other technologies.

The results of the analysis of the integration of support technologies for people with visual disabilities are shown in Table 1. The features that have been considered show that the basis of the systems is BCI and an EGG. The type of potential visual or auditory evoked stimulus depends on the disability in question. Finally, AC was considered as a field that allows identifying the affective state of a person. 

**Figure 2:**
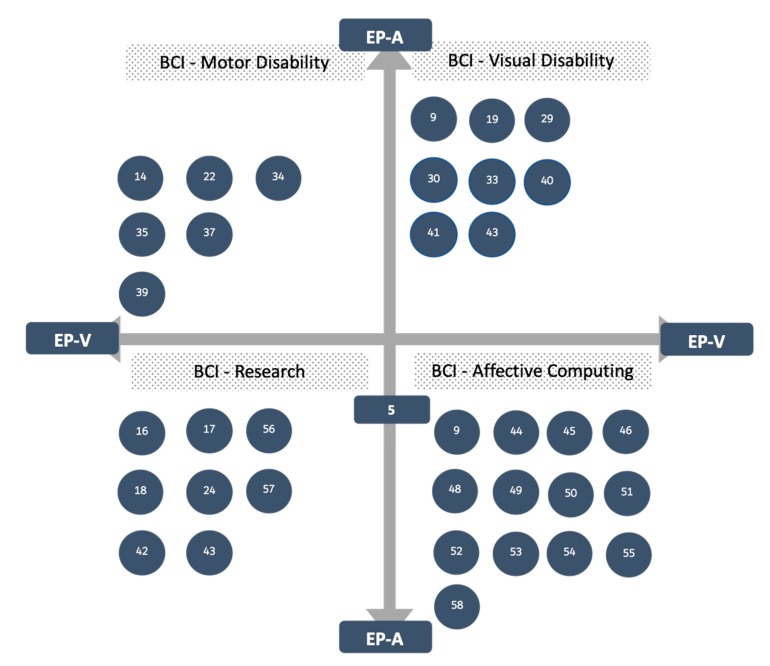
Trends in BCI and AC for people with disabilities.

Figure 2 concentrates the works reviewed in the field of investigation. The results show that the efforts performed to implement BCI systems and how to detect the affective state of a person with a visual disability still requires work.

## 5. Discussion

The answer to the first proposed question is discussed below: Why is it important to study the emotions of a person with a visual disability? Emotions are the way in which a person expresses their feelings—joy, anger, sadness, pleasure, etc.—before a certain situation or stimulus. However, this is difficult for individuals with a disability because they are not able to interact naturally. People with visual disabilities commonly require an intermediary that allows them to recognize and interact in the environment around them.

Affective computing has been shown to be applicable in the treatment of disorders such as autism, Asperger syndrome or depression, as well as in the recognition of stress and its mitigation. The study of affective states of a person with visual disabilities could be useful as a virtual assistant, which allows this type of person to express, recognize and interpret their emotions to improve their interaction with the environment, without the need to depend on someone else.

Although there are several ways of recognizing a person’s emotions, either through facial expressions, speech or bio-sensors, the study of brain signals by means of affective computation and a BCI is the main object of investigation of this work.

As indicated by Pantic et al. in [59] human–computer interaction should include the ability to recognize the affective states of users, to make systems more human, effective and efficient.

Regarding the second question stated in this paper: “Can affective computing obtain information of interest to represent the emotions of a person with a visual disability?”; to the best of our knowledge, the results do not show evidence of the integration of BCI and AC for detection of emotions in people with visual disabilities. However, to improve the efficiency in the interaction between the human and the computer, affective computation plays an important role; it could provide people with visual disabilities a new experience with the use of technology, through the detection of their emotions. Therefore, the authors identify that there is still a motivation to continue exploring areas that integrate affective computing, BCI systems and visual disabilities.

Based on the related research and on the results reported and analyzed, our manuscript shows that a BCI gives the opportunity for people with or without disabilities to communicate and interact with their environment through the interpretation of their brain signals. Under this approach, a BCI system widely used in the interpretation of brain signals can be based on a visual stimulus as a trigger; however, in people with visual disabilities, a BCI based on visual information is not entirely useful, which makes it necessary to move from visual stimuli to auditory stimuli in order to adjust the system to the needs of these people.

Emotions represent the affective state of a person and are expressions of mental states, given as a response to the stimuli produced in the environment. In addition, emotions influence the perception, communication and decision-making of people with or without disabilities. People with visual disabilities require auditory stimuli due to their condition. There are works related to the field of AC and BCI, which have positively reported the possibility of recognizing the affective states of a person who has been stimulated in an auditory way. 

**Figure 3:**
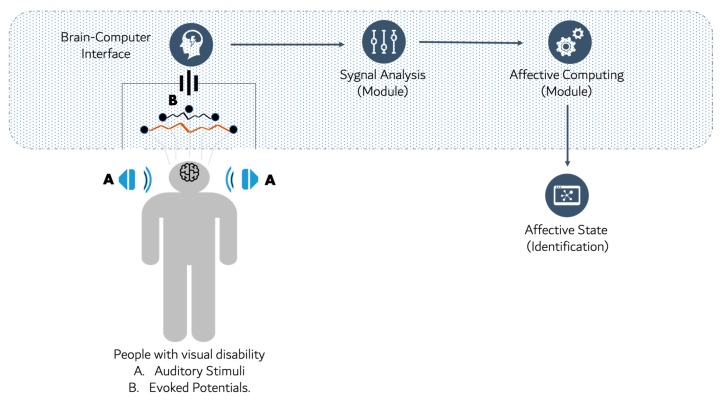
Integration of a BCI and AC for the detection of emotions in people with a visual disability.

Based on the observed lack of the related works and trends on the integration of BCI systems for the detection of the affective states of a person with a visual disability, the authors propose a framework for covering this gap. Our proposal is visualized in Figure 3.

The modules that compose our proposal are: (1) auditory stimulation of a person with visual disabilities; (2) use of a BCI to obtain the brain signals given by the evoked potentials; (3) an offline module to analyze the data set of the brain signals; (4) apply techniques for the extraction and classification of emotions, to finally pass to the module; (5) the identification of the affective state of the person with a visual disability.

There is also some research that identifies the emotions of people through physiological signals, as proposed by Healey et al. [60]. In this case, the authors mention that it is possible to recognize emotions using heart rate or muscle activity. 

In [61], Hamdi et al. evaluated the emotional states of a person using human–machine interfaces and biofeedback sensors. Their work evaluated the data in real-time, defined as a behavioral engine to allow a realistic multimodal dialogue between an incorporated conversational agent and the person.

Kousarrizi et al. mentioned that detecting artifacts produced in electroencephalography data by muscle activity, eye blinks and electrical noise is a common and important problem in electroencephalography research [62]. Also, researchers and scientists must consider the needs of users during the design and testing of BCI systems [63].

In BCI systems, users explicitly manipulate their brain activity instead of using motor movements to produce signals that can be used to control computers or communication devices [64]. 

A BCI offers people the opportunity to increase their capacities by providing a new bond of interaction with the outside world and is especially relevant as an aid to paralyzed people [65]. A BCI system provides people with visual, motor, severe motor or basic communication abilities with the ability to express their wishes, emotions or to communicate and even operate external devices [66].

## 6. Conclusions

The results of this review show that the efforts made in this area have implemented BCI based on auditory stimulation for people with visual disabilities. On the other hand, the affective computing has detected emotional states in people who do not have a visual disability, however, the implementation of a BCI using auditory stimuli in conjunction with affective computing, for the detection of emotions in people with a visual disability, still has not been proven. Therefore, the authors consider that the use of a BCI and AC for such individuals should be evaluated and they propose a framework architecture for integrating these areas.

A BCI provides another method of communication for those who have difficulty communicating with the outside world [67], researchers have used BCI technology to build systems that allow communication between the brain and the computer through brain signals.

The construction of robust and useful BCI models from accumulated biological knowledge and available data is a major challenge, as are the associated technical problems [68]. The needs of people with visual disabilities are greater every day. Technology will continue to make an impact on the lives of people with visual disabilities in ways that were not possible before [69]. In this sense, future research is needed in several areas, in addition to developing high performance BCI systems to allow people with needs to perform activities of daily living [70]. 

Therefore, research that is functional, and not just experimental, is a priority for people with visual disabilities as it could enable them to live a new experience with the use of technology. The priority goal was to improve the experience with technology and promote the integration of people with visual disabilities into society.

In future work the authors aim to implement the proposed framework in order to test its impact on people with visual disabilities. In addition, other future lines of research should be focused on the effect of audiovisual stimulation in healthy people and auditory stimulation in people with visual disabilities, in order to offer a similar experience with the use of technology. In the same way, further research is required in the response to the evoked potentials from a person with visual disabilities based on auditory stimulation. It is necessary to continue evaluating the effects that occur during these types of experiments in people with visual disabilities and evaluate the results in comparison to other types of stimuli presented to people without disabilities; that is, systems that adapt to the degree of stimulation required by people with visual disabilities. Another aspect that could be evaluated is the generation of adaptive recommendation systems for people with visual disabilities, which allow these people to select the audio according to their emotional states in real-time. Also, another future line would be the creation of an emotional virtual assistant for people with visual disabilities that identifies their emotions according to the environment in which they interact and gives them alternative communication and improvement of their affective state.

## Figures and Tables

**Table 1:** Brain–computer interface and affective computing for people with a visual disability.

Identifier	Year	Components	Description	Stimulus	Analysis	Accuracy	Extraction/Classification
[9] **Hamdi et al.**	2012	BCI, EEG, AC	Recognition of emotions through a BCI and a heart rate sensor	Visual	Online	Positive	Analysis of variance (ANOVA)
[14] **Pattnaik et al.**	2018	BCI, EEG	BCI for the classification of the movements of the left hand and the right hand	Visual	Online	Positive	Discrete wavelet transform (DWT)
[16] **Minguillon et al.**	2017	BCI, EEG	Identification of EEG noise produced by endogenous and exogenous causes	--	Offline	--	--
[17] **Jafarifarmad et al.**	2013	BCI, EEG	Extraction of noise-free features for EEG previously recorded	--	Offline	Positive	Functional-link neural network (FLN), adaptive radial basis function networks (RBFN)
[18] **Arvaneh et al.**	2011	BCI, EEG	Algorithm for EEG channel selection	Auditory/Visual	Offline	+10%	Sparse common spatial pattern (SCSP)
[19] **Kübler et al.**	2009	BCI, EEG, EP	BCI-controlled auditory event-related potential	Auditory	Online	--	Stepwise linear discriminant analysis method (SWLDA), Fisher’s linear discriminant (FLD)
[22] **Utsumi et al.**	2018	BCI, EEG	BCI for patients with DMD (Duchenne muscular dystrophy) based on the P300	Visual	Offline	71.6%–80.6%	Fisher’s linear discriminant analysis
[24] **Chi et al.**	2012	BCI, EEG	Analysis of dry and non-contact electrodes for a BCI	Auditory/Visual	Online	Positive	Canonical correlation analysis (CCA)
[29] **Sarwar et al.**	2010	BCI, EEG	Non-invasive BCI to convert images into signals for the optic nerve	Visual	Online	Positive	--
[30] **Guo et al.**	2010	BCI, EEG	A brain computer–auditory interface, using the mental response	Auditory	Offline	85%	Fisher discriminant analysis (FLD), support vector machine (SVM)
[33] **Klobassa et al.**	2009	BCI, EEG, EP	BCI based on P300	Auditory	Offline	50%–75%	Stepwise linear discriminant analysis method (SWLDA)
[34] **Sellers et al.**	2014	BCI, EEG	BCI non-invasive for communication of messages from people with motor disabilities	Visual	Online	Positive	Stepwise linear discriminant analysis method (SWLDA)
[35] **Okahara et al.**	2017	BCI, EEG	BCI based on P300 for patients with spinocerebellar ataxia (SCA)	Visual	Offline	82.9%–83.2%	Fisher’s linear discriminant analysis
[37] **Blasco et al.**	2012	BCI, EEG, AC	BCI based on EEG, for people with disabilities	Visual	Online	Positive	Stepwise linear discriminant analysis (SWLDA)
[39] **Hill et al.**	2012	BCI, EEG	BCI for completely paralyzed people, based on auditory stimuli	Auditory	Online	76%–96%	Contrast between stimuli
[40] **Suwa et al.**	2012	BCI, EEG, EP	BCI that uses the P300 and P100 responses	Auditory	Online	78%	Support vector machine (SVM)
[41] **Yin et al.**	2015	BCI, EEG, EP	Bimodal brain–computer interface	Auditory/Tactile	Online	+45.43–+51.05%	Bayesian linear discriminant analysis (BLDA)
[42] **Collinger et al.**	2013	BCI, EEG	Invasive brain–computer interface for neurological control	Visual	Online	Positive	--
[43] **Wang et al.**	2010	BCI, EEG	Portable and wireless brain–computer interface	Visual	Online	95.9%	Fast Fourier transform (FFT)
[44] **Daly et al.**	2018	BCI, EEG, AC	Analysis of brain signals for the detection of a person’s affective state	Auditory	Online	Positive	Support vector machine (SVM)
[45] **Williams et al.**	2017	BCI, EEG, AC	System for the generation of music dependent on the affective state of a person	Auditory	Online	Positive	--
[46] **Murugappan et al.**	2011	BCI, EEG, AC	Evaluation of the emotions of a person, using an EEG and auditory stimuli	Auditory/Visual	Offline	79.17%–83.04%	Surface laplacian filtering, wavelet transform (WT), linear classifiers
[48] **Khosrowabadi**	2010	BCI, EEG, AC	System for the detection of emotions based on EEG	Auditory/Visual	Offline	84.5%	The k-nearest neighbor classifier (KNN)
[49] **Mühl et al.**	2011	BCI, EEG, AC	Affective BCI using a person’s affective responses	Auditory/Visual	Online	--	A Gaussian naive Bayes classifier
[50] **Pentratonakis et al.**	2010	BCI, EEG, AC	Recognition of emotions through the study of EEG	Visual	Offline	62.3%–83.33%	K-nearest neighbor (KNN), quadratic discriminant analysis, support vector machine (SVM)
[51] **Nie et al.**	2011	BCI, EEG, AC	Classification of positive or negative emotions, studying an EEG	Visual	Offline	87.53%	Support vector machine (SVM)
[52] **Hsu et al.**	2015	BCI, EEG, AC	BCI non-invasive for the recognition of the emotions produced by music	Visual	Online	Positive	Artificial neural network model (ANN)
[53] **Byun et al.**	2017	BCI, EEG, AC	Classification of a person’s emotions using an EEG	Auditory	Offline	Positive	Band-pass filter
[54,55] **Sourina & Liu**	2013	BCI, EEG, AC	Algorithm of recognition of emotions in real-time, for sensitive interfaces	Visual	Online	Positive	Support vector machine (SVM)
[58] **Tseng et al.**	2015	BCI, EEG	Intelligent multimedia controller based on BCI	Auditory	Online	Positive	Fast Fourier transform (FFT)
[56] **Xu et al.**	2013	BCI, EEG	Performance of an auditory BCI based on related evoked potentials	Auditory	Online	+4%–+6%	Support vector machine (SVM)
[57] **Koelstra et al.**	2012	BCI, EEG	A database for the analysis of emotions	Visual	Offline	--	High-pass filter, analysis of variance (ANOVA)

Terms referred to in Table 1: BCI (brain–computer Interface), EEG (electroencephalogram), AC (affective computing), evoked potentials (EP).
